# Detection of Group B Streptococcus (GBS) from Antenatal Screening, Maternal GBS Colonization and Incidence of Early-Onset Neonatal Disease (GBS-EOD): A National Survey, December 2022 to February 2023, Italy

**DOI:** 10.3390/microorganisms13071438

**Published:** 2025-06-20

**Authors:** Michela Sabbatucci, Pierangelo Clerici, Roberta Creti

**Affiliations:** 1 Unit 2, Directorate General of Health Emergencies, Department of Prevention, Research and Health Emergencies, Ministry of Health, 00144 Rome, Italy; m.sabbatucci@sanita.it; 2 Italian Clinical Microbiologists Association (AMCLI), 20159 Milano, Italy; pierclerici@gmail.com; 3 Antibiotic-Resistance and Special Pathogens Unit, Department of Infectious Diseases, Istituto Superiore di Sanità, 00161 Rome, Italy

**Keywords:** group B streptococcus, GBS, *Streptococcus agalactiae*, neonatal GBS disease, antibiotic resistance, antenatal GBS screening

## Abstract

Invasive neonatal GBS infections constitute a major cause of sepsis and meningitis in Western countries. Vaginal/rectal GBS colonization during pregnancy is the main risk factor for the development of early-onset infections (GBS-EOD) in newborn by vertical transmission at birth, in addition to prematurity and stillbirth. In Italy, intrapartum antibiotic prophylaxis (IAP) to prevent GBS-EOD is offered to pregnant women who tested as GBS-positive in late pregnancy. Passive surveillance in Italy showed that a non-negligible number of GBS-EOD cases (about 50%) occurred from GBS-negative pregnant women. This finding prompted the launch of a national online survey from 15 December 2022 to 12 February 2023 to investigate the microbiological procedures followed for GBS identification in Italian public and private microbiology laboratories, the prevalence of maternal GBS colonization, and the incidence of GBS-EOD cases. The survey results demonstrated that national guidelines for the prevention of EOD-GBS cases as well as harmonization of microbiological methodologies for GBS identification in the antenatal screening are needed.

## 1. Introduction

Early-onset neonatal group B streptococcus disease (GBS-EOD) occurs in the first week of life, mostly within 6 h after birth, presenting with pneumonia, sepsis and, to a lesser extent, meningitis [1,2,3]. GBS is part of the gastro-intestinal and vaginal microbiota in 10–30% of healthy pregnant women. Maternal colonization may be transitory, intermittent, or persistent. Maternal GBS vaginal/rectal colonization is the main risk factor for the development of GBS-EOD by bacterial vertical transmission during labour or after rupture of membranes [4,5,6,7].

In the absence of any prevention strategy, around half of the newborns from GBS-colonized mothers are in turn colonized at the gastrointestinal and respiratory level; of colonized neonates, 1–2% develop invasive disease [8]. The infection can also occur even before birth by ascending migration of the bacterium towards the uterus in the placental sac, causing intrauterine death, preterm labour or the birth of an already septic newborn. GBS-EOD has also been correlated with a higher probability of developing necrotizing enterocolitis compared with GBS-negative newborns [9].

Antenatal GBS screening (AGBSS) is recommended for all pregnant women at 36 to 37 weeks of gestation by Italian guidelines [10,11]. Performed on schedule (no more than five weeks before delivery), using the correct procedure (vaginal and rectal sampling), and with the appropriate culture procedure (enrichment in broth before inoculation on selective medium), the results of AGBSS are extremely reliable [12,13,14]. Unfortunately, specific instructions on the microbiological procedure for the identification of GBS from the vaginal/rectal swabs are not present in the Italian guidelines.

Intrapartum antibiotic prophylaxis (IAP) by intravenous administration of penicillin/ampicillin to pregnant women who tested positive at AGBSS is a consolidated strategy to prevent vertical transmission in high prevalence areas. In the case of penicillin allergic women, clindamycin can be the second-line antimicrobial for IAP [15,16,17,18,19,20,21,22,23].

A global systematic review estimated the pooled incidence of invasive GBS disease in infants in the period 2000–2017 as 0.49 per 1000 live births, with the highest value in Africa (1.12) and the lowest in Asia (0.30). Mostly, GBS belonged to the serotype III (61.5%) [24]. In the United States, incidence rates of GBS-EOD have declined (from 1.7 to 0.25 cases per 1000 live births between 1990s and 2010) due to both adoption of GBS-prevention guidelines and subsequent introduction of universal antenatal screening, in addition to improvements in obstetric practices [25].

Despite a relatively low incidence of invasive neonatal GBS infection, the risk of death or severe complication remains high; in high-income countries (HIC) mortality rates still range around 4–10% [26].

In Italy, since 2003, an active surveillance system is in place in the Emilia-Romagna Region [27]. In addition, birth centres from the rest of the country report cases of invasive neonatal GBS infections and send the bacterial isolates to the National Institute of Health (Istituto Superiore di Sanità, ISS) on a voluntary basis, allowing the monitoring of circulating serotypes and rate of antibiotic resistance [28,29]. Based on incidence data calculated in the period 2013–2016 from Emilia-Romagna (0.20 GBS-EOD cases per 1000 live births), in Italy, at least 100 cases of GBS-EOD are estimated per year.

In Italy, IAP to prevent GBS-EOD is offered to pregnant women who tested positive on the AGBSS [10,11]. Intrapartum beta-lactam antibiotics are associated with a decreased risk of GBS-EOD, mostly if within 4 h of birth. The national passive surveillance conducted by ISS showed that about half of GBS-EOD cases occurred from GBS-negative pregnant women [28,29]. Indeed, a previous nation-wide investigation indicated that AGBSS was performed following different protocols by the microbiology laboratories, possibly leading to false-negative AGBSS results and a higher risk of GBS-EOD [30].

Considering that the GBS identification in antenatal screening can impact the appropriateness of IAP use to reduce GBS-EOD cases, a national online survey from 15 December 2022 to 12 February 2023 was conducted to investigate the microbiological procedures followed for GBS identification in Italian public and private microbiology laboratories, the prevalence of maternal GBS colonization, the incidence of neonatal GBS-EOD cases and the rate of antimicrobial resistance.

## 2. Materials and Methods

### 2.1. Study Design

A national survey was promoted by the Istituto Superiore di Sanità, in collaboration with the Ministry of Health and the Italian Clinical Microbiologists Association (AMCLI).

In particular, the notice was sent by the Ministry of Health to regional health offices, which distributed it to their list. In addition, the Clinical Microbiologists Association promoted the initiative among its members who work in both public and private laboratories.

The survey was distributed through an online password-protected questionnaire from 15 December 2022 to 12 February 2023, available on Survio (Cleverbridge GmbH, Colonia, Germania).

The questionnaire included 22 questions with the aim to collect information on GBS-EOD neonatal invasive infection, data on maternal GBS vaginal/rectal colonization, the microbiological method used for performing AGBSS, and antimicrobial resistance for the years 2018–2022 (Table S1).

We applied the GBS-EOD case definition, as an invasive neonatal infection occurring within 6 days of birth with GBS being isolated from blood and/or cerebrospinal fluid.

Data were analyzed by R Studio statistical program version 4.4.0. GBS-EOD incidence was calculated by dividing the number of cases by the number of births registered in the participating facilities per year.

The prevalence of maternal vaginal/rectal GBS colonization was calculated by dividing the number of GBS-positive swabs by the total number of swabs performed in one year in participating facilities.

Only for the seven regions/autonomous provinces that provided complete data (AP Bolzano, Friuli Venezia Giulia, Lombardia, Marche, Sicilia and Veneto), we calculated both the overall 5-year and year-by-year prevalence of GBS-EOD cases born to GBS-negative mothers by dividing their number by a denominator calculated by subtracting the number of maternal swabs tested positive from the total number of those performed.

### 2.2. Ethical Approval

The information provided by the individual participating centres complied with local legislation on data protection and privacy. Ethical approval was not required for the study, as it involved retrospective analysis of anonymized data.

## 3. Results

### 3.1. Compliance to the Online Survey

**Figure 1:**
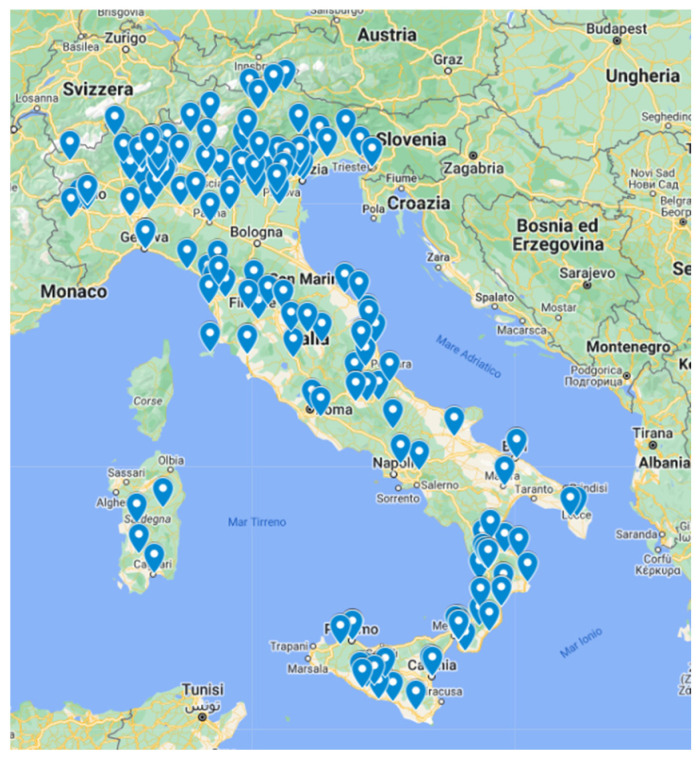
Map of the 161 locations of the centres (*N* = 218) that participated to the national survey on Group B Streptococcus (GBS), Italy, December 2022 to February 2023.

All the 19 Italian regions (R) and the 2 autonomous provinces (AP) participated in the survey by completing the online questionnaire, with a total of 218 participating centres (Figure 1) from 78 provinces and 161 towns (Table 1). Each participating centre completed the questionnaire only once.

Almost half of the participating centres were distributed in the North of the country (North-West, 31.1%; North-East, 17.6%), while 19.8% were located in the Centre, 18.9% in the South, and 12.6% on the two islands.

Table 1 showed the proportion of compiled questionnaires per R/AP, along with the population registered in the year 2023 in each R/AP.

In Italy, about one-sixth of residents live in Lombardia, which therefore represents the most populous region in the country, and contributed to the majority (22.0%) of the questionnaires.

Almost all participants compiled the questionnaire through the link provided (95.0%), while few used the QR code (2.3%) or provided the pdf file by email (2.7%).

### 3.2. GBS-EOD Incidence over Time

**Figure 2:**
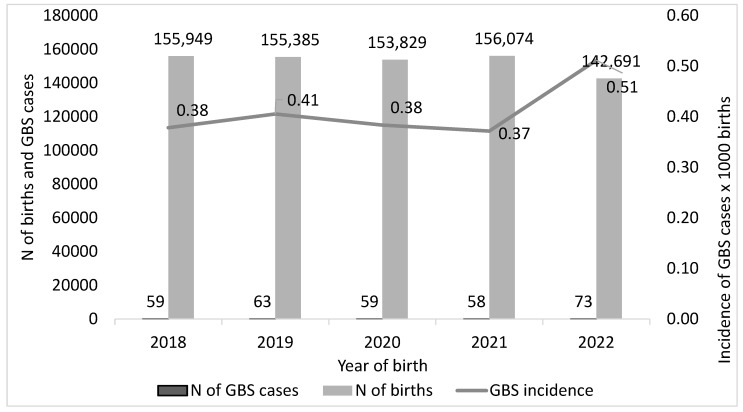
Incidence of neonatal Group B Streptococcus (GBS, cases per 1000 live births) in the years 2018–2022, Italy.

For the calculation of the incidence (number of neonatal GBS cases/number of annual births) we excluded all situations where the numerator or denominator were not available. Therefore, we considered 312 GBS-EOD cases (from 59 cases in 2018 to 73 in 2022) and 763,928 births, and calculated an overall GBS-EOD incidence of 0.41 per 1000 births during the study period. Incidence increased by 34.2%, from 0.38 in 2018 to 0.51 in 2022 (Figure 2).

Of note, the decreasing number of births declared each year by the participating centres mirrored the trend reported by the National Institute of Statistics (Istat) between 2018 and 2022. Our survey collected data on 35.5% of national births registered by Istat in 2018, 37.0% in 2019, 38.0% in 2020, 39.0% in 2021, and 36.3% in 2022 (Table 2).

**Figure 3:**
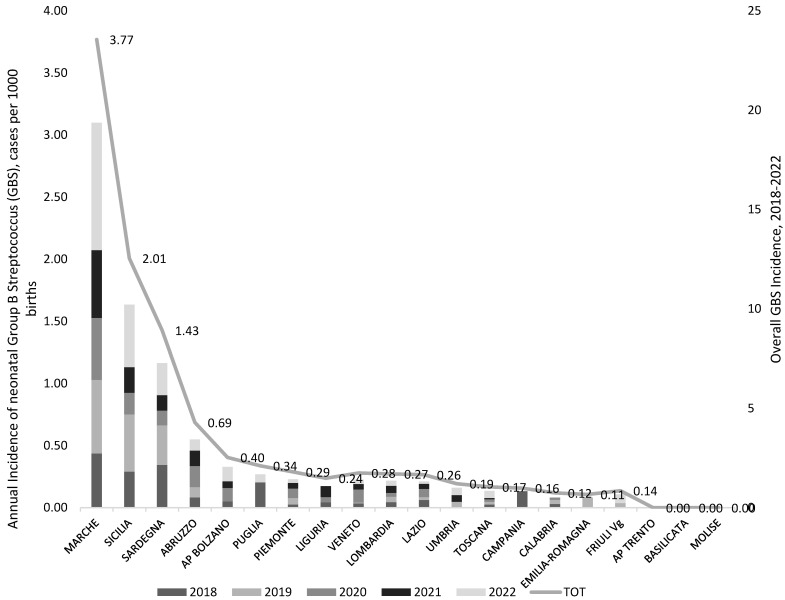
Incidence of GBS-EOD (GBS, cases per 1000 live births) by region/autonomous province (R/AP) and year of birth (2018–2022), national survey from December 2022 to February 2023, Italy. The region Valle D’Aosta was excluded from the graph as it reported no data.

The GBS-EOD incidence calculated for each region from the year 2018 to the year 2022 is reported in Figure 3.

Case incidence showed great variability both between regions and within the same region year after year. This does not necessarily reflect the real trend of neonatal GBS incidence over time.

### 3.3. Maternal GBS Colonization

**Figure 4:**
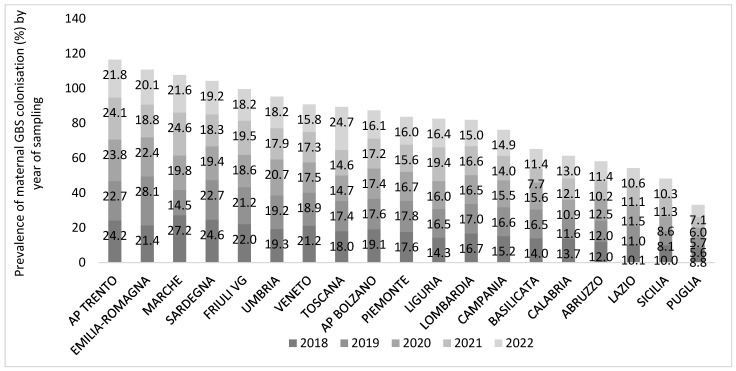
Prevalence (%) of maternal Group B Streptococcus (GBS) colonization by participating region/PA and year of sampling, national survey from December 2022 to February 2023, Italy. The regions Molise and Valle D’Aosta were excluded from the graph because of uncomplete data.

Prevalence of maternal GBS colonization varied largely between regions and year of sampling, ranging from 5.6% in Puglia in 2019 to 28.1% in Emilia Romagna in the same year. We excluded from this analysis two regions (Molise and Valle D’Aosta) for which complete data were not available (Figure 4). The mean national prevalence of maternal GBS colonization decreased by 1.3% in the reference period from 17.2% in 2018 to 15.9% in 2022 (median values 17.6% in 2018, 16.0% in 2022). Some regions had an overall mean prevalence of maternal GBS colonization resulting four times higher (over 23%) than the lowest regional one (Puglia, 6.6%) in the time period considered. Seven R/APs experienced a drastic reduction in the regional prevalence through the years (Basilicata, AP Bolzano, Friuli Venezia Giulia, Marche, Sardegna, AP Trento, Veneto) while two regions (Liguria and Toscana) presented a positive trend, and the others had no considerable changes (Figure 4).

A weak negative correlation (Pearson coefficient R = −0.246; R^2^ = 0.061) was found between the size of the regional population and the GBS-colonized maternal population: 6.1% of the variation in the dependent variable (GBS-colonized maternal population) is explained by the independent variable (size of the regional population).

Similarly, a weak negative correlation (R = −0.174) was found between the number of facilities participating in the survey and the GBS-colonized maternal population: only 5.6% of the variation in the GBS maternal prevalence is explained by the number of facilities.

Among the four R/PA with the highest overall maternal prevalence (over 20% in the period 2018–2022, AP Trento, Emilia-Romagna, Marche, Sardegna), Marche and Sardegna reported also the highest neonatal GBS-EOD incidence (3.77 and 1.43 per 1000 newborns, respectively). On the contrary, the region Sicilia, despite the low maternal colonization prevalence (9.6%), presented a high neonatal GBS-EOD incidence (2.13 per 1000 newborns).

All the other participating Rs reported a neonatal incidence below 0.7 per 1000 newborns.

### 3.4. Detection and Identification of GBS at the Antenatal Screening

Assuming a ratio of one mother/one newborn, during the study period, the proportion of pregnant women who undertook the AGBSS were 144,527/155,949 (92.7%) in the year 2018, 153,987/155,385 (99.1%) in the year 2019, 142,780/153,829 (92.8%) in the year 2020, and 148,880/156,074 (95.4%) in the year 2021. In 2022, we asked the participating centres to report the number of swabs and births until 15 December. Therefore, this could be the cause for the number of swabs resulting in a higher value than the number of births (145,682/142,691 in the year 2022).

**Figure 5:**
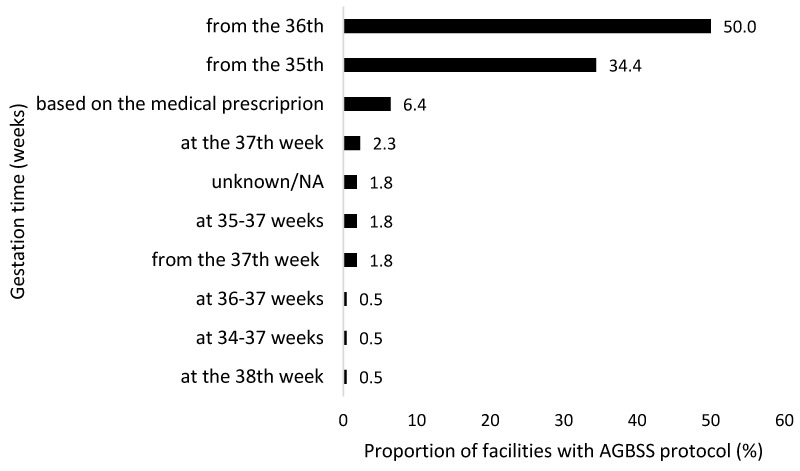
Timing (week of gestation) for antenatal Group B Streptococcus screening (AGBSS) as declared by the centres participating to the national survey, December 2022 to February 2023, Italy (*N* = 198).

A total of 186 (85.3%) participating centres confirmed the availability of a local microbiological protocol for AGBSS, while 32 (14.7%) facilities reported the absence of a shared protocol. Mostly, the AGBSS was performed starting from the 36th (55.6%) or 35th (39.4%) week of gestation, or on medical prescription (7.1%) (Figure 5).

Almost all centres performed both vaginal and rectal swabs (98.2%), while a minority performed vaginal swabbing only (1.4%).

**Figure 6:**
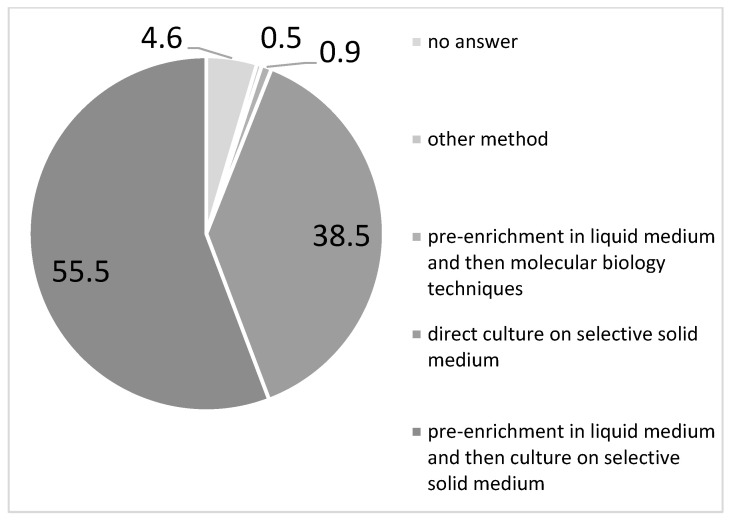
Type (%) of microbiological tests performed for antenatal Group B Streptococcus screening (AGBSS) reported in a national survey on GBS by the participating centres (*N* = 218), December 2022 to February 2023, Italy.

Regarding the microbiological method used, 95.4% of participants provided information. Most of the respondents declared they performed pre-enrichment in liquid medium followed by culture on selective solid medium (55.5%) or molecular biology techniques (0.9%), while over one third of them (38.5%) used culture on selective solid medium alone without any pre-enrichment of the sample. Few of them performed other methods (0.5%) (Figure 6). A total of 1.4% of the responding facilities sent the samples to the microbiological laboratory.

The CDC-recommended pre-enrichment broth step was followed more frequently by hospital laboratories (63.5%) than by private laboratories (49.2%).

Notably, in the two R/APs that showed no GBS-EOD cases or very low incidence and high prevalence of maternal colonization (AP Trento and Emilia-Romagna, respectively; Figure 3 and Figure 4), all the participating centres declared to perform pre-enrichment culture.

Sicilia and Abruzzo regions, although with some of the lowest maternal prevalence (9.6% and 11%, respectively), registered the second (2.01 per 1000 births) and the third (0.69 per 1000 births) highest GBS neonatal incidence. Notably, just over half (56.5% and 66.7%, respectively) of the 29 responding facilities from both these two R performed pre-enrichment culture.

### 3.5. GBS-EOD Neonates Born to Mothers Negative or Positive for AGBSS

Only five R and one A/P provided complete data on the number of GBS-EOD neonates born to mothers negative for AGBSS. In total, 93 cases were reported on a total of 231 GBS-EOD cases that occurred in those regions, corresponding to an incidence of 40.2%, slightly less than that observed in the national passive surveillance in the years 2015–2019 [29].

The prevalence of GBS-EOD cases from mothers who tested negative for AGBSS (93 GBS-EOD cases out of 336,801 women between 2018 and 2022) was 0.028% with a great variability range among the six regions (Bolzano: 0.037%, Friuli Venezia Giulia: 0.0086%, Lombardia: 0.018%, Sicilia: 0.054%, Veneto 0.0019%, Marche: 0.72%).

The Pearson correlation coefficient 0.10 showed that there was not linear correlation between GBS maternal colonization and neonatal incidence in the 19 R/AP considered for which both data were available. On the contrary, we highlighted strong and positive linear correlation (Pearson index = 0.9) between the prevalence of GBS-EOD cases born from mothers who tested GBS-negative and the incidence of GBS newborns.

### 3.6. Antibiotic Resistance

Almost half (40.3%) of the participating centres surveyed (*N* = 208) used the microbiological method to test the inducible resistance to clindamycin When asked about the number of clindamycin-resistant GBS strains and the total number of tests performed between 2020 and 2022, a total of 2255 GBS strains out of 19,306 tests performed were resistant to clindamycin in the reference period, with an overall 3-year prevalence of 11.7% (11.1% in 2020, 12.4% in 2021, 11.6% in 2022). 

**Figure 7:**
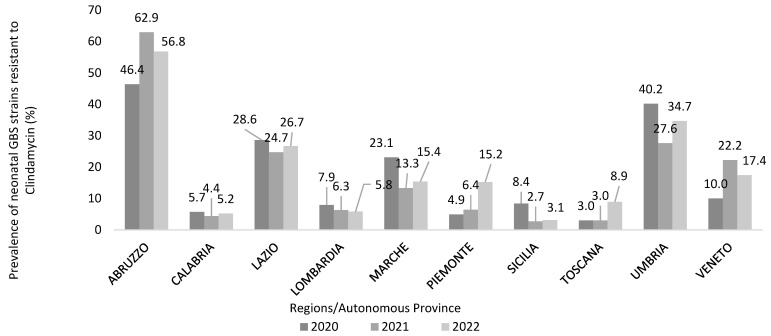
Prevalence of neonatal Group B Streptococcus (GBS) strains inducibly resistant to clindamycin (%) in a three-year period, calculated by a national survey on GBS through the participating healthcare facilities (*N* = 218), December 2022 to February 2023, Italy.

Nevertheless, the incidence of clindamycin resistance varied largely between regions reaching very alarming levels in some of them (Abruzzo, Umbria) (Figure 7).

Moreover, in the questionnaire we asked whether the resistance of GBS isolates to high levels of aminoglycosides was routinely tested. Based on the information provided by 205 relevant answers, 34 (16.6%) respondents confirmed this microbiological service.

High-level gentamicin resistance was tested by 38 (19.3%) out of 197 respondents. Of them, 16.7% indicated the use of the European Committee on Antimicrobial Susceptibility Testing (EUCAST) breakpoints [31] for the gentamicin susceptibility testing, while 1.1% indicated the Clinical and Laboratory Standards Institute (CLSI) breakpoints [32].

## 4. Discussion

In this study, we collected data on GBS-EOD and GBS colonization in late pregnancy, as well as information on the microbiological method used for the detection and identification of GBS in the antenatal screening in Italy between 2018 and 2022. The national survey was well participated in across the country, covering 35–39% of the number of newborns registered between 2018 and 2022 in Italy. Over half of the respondents took less than 20 min to complete the online questionnaire, revealing this tool as a good choice that can be used in the future.

During the reference period (2018–2022), the incidence of neonatal infection increased by 34.2%, while the prevalence of maternal GBS colonization decreased by 1.3%, with no geographical gradient. Overall, prevalence of maternal colonization registered high levels, ranging from 6.6% (Puglia) to 23.3% (AP Trento). We highlighted that just three Rs presented high levels of neonatal GBS-EOD incidence aligned with the highest prevalence of maternal colonization. Instead, in most cases, neonatal GBS-EOD incidence registered levels below 0.4 per 1000 births.

However, the Pearson correlation coefficient was not statistically significant, showing an absence of linear correlation between GBS maternal colonization and neonatal incidence. This unclear trend could be due to several factors other than the AGBSS microbiological method used locally and the administration of IAP, which we investigated in this study, such as infection prevention and control (IPC) practices applied at the time of delivery or after labour, diverse efficacy in microbiological detection, and local GBS epidemiology. Notably, the lowest maternal GBS prevalence could be due to absence of pre-enrichment practice in the microbiological GBS identification (false negative test).

A previous national survey conducted in the year 2012 [30] reported that the pre-enrichment broth step was performed by 58.8% of the 101 respondent microbiological laboratories. Unfortunately, our data demonstrated that this behaviour has not changed over time, and only 56.4% respondents in our survey apply the pre-enrichment step, with a higher compliance for hospital laboratories.

This finding posed a serious warning, because the sensitivity of GBS detection is strongly impacted by the pre-enrichment broth step, which is considered a key step for maximizing GBS identification [33,34,35]. Incubation in selective broth media prior to plating increases the sensitivity of screening methods by about two-fold compared to direct specimen plating by inhibiting the growth of enteric organisms which overgrow GBS, making detection difficult [14]. Even in the case of the use of nucleic acid amplification testing (NAAT) or other rapid techniques for processing of antepartum cultures, the recommended procedure includes an 18–24 h incubation step before performing the NAAT analysis [14,36,37,38]. To speed up the GBS identification, differential chromatic enrichment broths as Carrot Broth, Granada Liquid Biphasic broth can be used allowing the visual identification of GBS by an orange-red colour pigment production [39,40]. Nevertheless, non-hemolytic GBS strains (about 5%) do not produce pigment, so subcultures of all pigment-negative broth cultures to agar plates is recommended [41].

Another crucial step in the AGBSS method is the swabbing method. The processing of vaginal-rectal swab specimen enhances the GBS identification compared with sampling the vagina without a rectal culture [14,20,42,43,44,45,46]. Fortunately, our survey indicated that this recommendation has been perceived by microbiologists in our country, with only 1.4% of the respondents declaring that only the vaginal swab is performed. These data represent a significant improvement compared to the previous first national survey in 2012, in which only 66.4%, 61.2%, 43.3% of the microbiological laboratories (respectively, located in northern, central and southern Italy) performed the vaginal–rectal swabbing [30].

Our analysis showed high regional variability in the prevalence of GBS-EOD cases born from mothers who tested as GBS-negative; overall, one third (93 out of 312 cases) of GBS-EOD-infected children in the 5-year period considered were born from mothers who tested negative to AGBSS. We showed a strong and positive linear correlation (Pearson index = 0.9) between the prevalence of GBS-EOD cases in children from mothers who tested GBS-negative and the incidence of GBS newborns, indicating that missing maternal GBS identification due to false negative tests impact the incidence of cases. Overall, these factors could explain both the large variability in the regional prevalence of GBS-colonized pregnant women and the high proportion of GBS-EOD cases born from GBS-negative mothers [47,48,49], indicating the need for national prevention guidelines that can facilitate the harmonization of AGBSS protocols, including with regard to the microbiological method used and limit the GBS-EOD burden in Italy.

A total of 55.6% of respondents reported performing AGBSS starting “from the 36th week of gestation” and 39.4% “from the 35th week of gestation”. The Italian guidelines [10,11] recommend universal GBS screening between 36 0/7 and 37 6/7 weeks of gestation, and the most recent American College of Obstetricians and Gynecologists (ACOG) guideline [20] is now aligned with this timeframe, whereas the previous guideline recommended prenatal GBS screening from 35 0/7 weeks of gestation.

Our national survey demonstrated that this one-week forward shift in AGBSS needs to be further encouraged to ensure a high degree of accuracy in predicting GBS colonization status at birth.

Clindamycin may be the second-line antibiotic in IAP in penicillin-allergic women [20]. Based on our survey, inducible and constitutive resistance to clindamycin was routinely performed only by 41.6% of the participating hospitals. Moreover, it is unclear how this information is actually available to obstetricians during labour.

Clindamycin resistance in GBS occurs at an alarming rate in many countries [50,51]. The UK National Institute for Health and Care Excellence (NICE) guidelines have changed their recommendation to use clindamycin as an alternative for penicillin-allergic women in IAP [52]. In contrast, in the United States, clindamycin is still the second antibiotic of choice in the case of women allergic to penicillin with a high risk of anaphylaxis [20].

Our survey, as well as recent studies on the surveillance of invasive neonatal and infant GBS, and in GBS-colonized women [28,29,53,54,55], indicate that clindamycin resistant GBS has been steadily increasing at alarming rates.

In the absence of Italian guidelines addressing the antibiotic of second choice in IAP, our survey indicated that the use of clindamycin in penicillin allergic women should be carefully considered if an antibiotic susceptibility test is not available, because in one case out of three, the GBS strain colonizing the parturient can be clindamycin resistant.

Our survey also focused on the high-level gentamicin resistance (HLGR) rate in GBS. We are observing increasing resistance to this antibiotic in GBS [56,57]. Nevertheless, the clinical significance of the emergence of HLGR in GBS is still not clear. Gentamicin is not an antibiotic used in the case of GBS infection, but it is administered in combination with ampicillin as an initial empiric therapy for neonatal sepsis and meningitis because of enhanced bactericidal activity and to obtain wide-spectrum bacterial coverage [58,59,60,61].

High-level aminoglycoside resistance abrogates enhanced bactericidal activity and this can constitute an advantage also for GBS. Our survey confirmed the poor investigation of HLGR in GBS and difficulty in the choice of interpretative criteria for assessing HLGR in GBS that are lacking both in the CLSI and EUCAST guidelines. Only the Comitte de la Antibiogramme de la Societe Francaise de Microbiologie (CA-SFM/EUCAST) considers interpretative criteria for gentamicin and streptomycin for beta-haemolytic streptococci.

Our study has some limitations. The calculation of prevalence GBS-EOD cases from GBS negative mothers was performed indirectly and could, therefore, be distorted, mainly by two factors. One, the mother may not have given birth in the same region where she took the AGBSS. Second, her GBS colonization status may have changed at the time of birth.

When applied, we considered global population data as denominator but the coverage of the participating centres for each region was not total so, for some estimates, there may be an under-bias.

## 5. Conclusions

This study is the result of a survey on GBS-EOD and its prevention, never before conducted in Italy on such an extensive basis.

Our results indicate the urgent need for a national guideline for the harmonization of the microbiological protocol of AGBSS in order to maximize the current efforts for the prevention of GBS-EOD in our country.

## Figures and Tables

**Table 1:** Proportion (%) of compiled questionnaires from participating centres and towns per region (R) or autonomous province (AP); national survey on Group B Streptococcus (GBS), Italy, December 2022 to February 2023.

Region/Autonomous Province	Proportion of Compiled Questionnaires (%)	Participating Centres That Completed the Questionnaire (*N*)	Towns Where the Facilities Where Located (*N*)	Regional Population (1 January 2023)
Lombardia	22.0	48	31	9,976,509
Calabria	14.6	32	19	1,846,610
Sicilia	10.5	23	19	4,814,016
Veneto	11.0	24	20	4,849,553
Toscana	8.7	19	16	3,661,981
Piemonte	5.5	12	10	4,251,351
Marche	3.6	8	7	1,484,298
Abruzzo	2.8	6	5	1,272,627
Friuli-Venezia Giulia	2.8	6	4	1,194,248
Lazio	2.8	6	2	5,720,536
Liguria	2.8	6	3	1,507,636
Puglia	2.3	5	4	3,907,683
PA Bolzano	1.8	4	4	534,147
Sardegna	1.8	4	4	1,578,146
Umbria	1.8	4	4	856,407
Campania	1.4	3	3	5,609,536
PA Trento	1.4	3	1	542,996
Emilia-Romagna	0.9	2	2	4,437,578
Basilicata	0.5	1	1	537,577
Molise	0.5	1	1	290,636
Valle d’Aosta	0.5	1	1	123,130
Total	100	218	161	58,997,201

**Table 2:** Comparison of the numbers of births between ISTAT and our study.

N of Newborns Per Year, Italy	2018	2019	2020	2021	2022
data source: ISTAT	439,747	420,084	404,892	400,249	393,333
data source: GBS SURVEY total 763,928	155,949	155385	153,829	156,074	142,691
ratio ISTAT/SURVEY	2.8	2.7	2.6	2.6	2.8
proportion (%) SURVEY/ISTAT	35.5	37.0	38.0	39.0	36.3

## Data Availability

The raw data supporting the conclusions of this article will be made available by the authors on request.

## Supplementary Materials

The following supporting information can be downloaded at https://www.mdpi.com/article/10.3390/microorganisms13071438/s1. The questionnaire distributed is available as Table S1. It has been translated to English.
