# Supplementary material for: Detection of Group B Streptococcus (GBS) from Antenatal Screening, Maternal GBS Colonization and Incidence of Early-Onset Neonatal Disease (GBS-EOD): A National Survey, December 2022 to February 2023, Italy
Source: Microorganisms. 2025 Jun 20;13(7):1438. doi: 10.3390/microorganisms13071438 (PMC12299446; doi:10.3390/microorganisms13071438)
Supplement: Supplementary file 1 [file microorganisms-13-01438-s001.zip › Table S1.pdf]

QUESTIONNAIRE ON MICROBIOLOGICAL ASPECTS FOR THE PREVENTION OF NEONATAL INVASIVE INFECTION BY GROUP B STREPTOCOCCUS (Streptococcus agalactiae, GBS)
National survey, December 2022 to February 2023

Preliminay questions (1 to 6) for information on the participating Centre

1. Please, select the Region/Autonomous Province in which the respondent Centre is located
2. Please, insert the Province
3. Please, insert the zip code
4. Please, insert the town
5. Please insert the name of the participating Centre
6. If available, please provide a reference person details

7. How many cases of invasive early GBS infections (GBS-EOD) (intended as a bacterial isolate or positive molecular test from blood and/or cerebrospinal fluid) in newborns aged 0-6 days have occurred in your hospital in the years:
   2018
   2019
   2020
   2021
   2022 until December 15th

8. To calculate the GBS.EOD incidence, what was the number of births in the years:
   2018
   2019
   2020
   2021
   2022 until December 15th

9. Do you have an internal protocol for the antenal GBS screening?
   Yes
   No

10. Which national/international recommendations do you refer to for performing the antenatal GBS screening?
    text input field only

11. At what gestational age do you perform the antenatal GBS screening'
    starting fronm the 35th weeks
    starting from the 36th week
    other

12. What type of sample collection do you perform?
    only vaginal swabbig
    vaginal and rectal swabbing

13. After the swabbing, your microbiological protocol recommend to:
    to strike the swab on selective agar plate

to perform an pre-enrichment step before plating

14. describe in more detail the procedure used (types of media, temperature and incubation time, isolation, use of chromogenic media)
    text input field only

15. How many samples resulted positive for GBS and how many antenatal GBS screening tests have you performed in the years:
    2018
    2019
    2020
    2021
    2022 untile December 15th

16. In the case of unknown GBS status of the parturient, do you perfom a GBS intrapartum test?
    Yes
    No

17. What is the incidence of GBS-EOD cases born to mothers who tested negative for the antenatal GBS screening over the years:
    2018
    2019
    2020
    2021
    2022 untile December 15th

18. Do you test for inducible clindamycin resistance of GBS isolated from the antenatal screening?
    Yes
    No

19. if you have answered yes to the previous question, please indicate the number of inducible clindamycin resistant GBS and the total number of the tests performed in the years:
    2020
    2021
    2022 untile December 15th

20. Do you routinely test the high-level aminoglycoside resistance of GBS?
    Yes
    No

21. If yes, do you perform the gentamicin susceptibility test?

22. What breakpoint for the gentamicin susceptibility you refer to?
